# Assessing the quality of the care offer for people with personality disorders in Italy: the QUADIM project. A multicentre research based on the database of use of Mental Health services

**DOI:** 10.1186/s13033-023-00603-9

**Published:** 2023-10-13

**Authors:** Michele Sanza, Matteo Monzio Compagnoni, Giulia Caggiu, Liliana Allevi, Angelo Barbato, Jeannette Campa, Flavia Carle, Barbara D’avanzo, Teresa Di Fiandra, Lucia Ferrara, Andrea Gaddini, Alessio Saponaro, Salvatore Scondotto, Valeria D Tozzi, Stefano Lorusso, Cristina Giordani, Giovanni Corrao, Antonio Lora

**Affiliations:** 1 Department of Mental Health and Addiction Disorders Forlì-Cesena, AUSL Romagna, Cesena, Italy; 2 Unit of Biostatistics, Epidemiology and Public Health, Department of Statistics and Quantitative Methods, University of Milano-Bicocca, Milan, Italy; 3 National Centre for Healthcare Research and Pharmacoepidemiology, University of Milano-Bicocca, Milan, Italy; 4 Department of Mental Health and Addiction Services, ASST Lecco, Lecco, Italy; 5 Department of Health Policy, Istituto di Ricerche Farmacologiche Mario Negri IRCCS, Milano, Italy; 6 Addiction Unit, AUSL Romagna, Cesena, Italy; 7 Center of Epidemiology and Biostatistics, Polytechnic University of Marche, Ancona, Italy; 8 Psychologist, previously General Directorate for Health Prevention, Ministry of Health, Rome, Italy; 9 Centre of Research on Health and Social Care Management, CERGAS SDA Bocconi School of Management (Bocconi University, Milan, Italy; 10 Agency for Public Health, Lazio Region, Rome, Italy; 11 General Directorate of Health and Social Policies, Emilia-Romagna Region, Bologna, Italy; 12 Department of Health Services and Epidemiological Observatory, Regional Health Authority, Sicily Region, Palermo, Italy; 13 Department of Health Planning, Italian Health Ministry, Rome, Italy

**Keywords:** Healthcare utilization databases, Personality disorders, Quality of mental healthcare, Treatment gap, Clinical pathways, Mental healthcare, Real-world, Healthcare research, Public health, Healthcare services

## Abstract

**Background:**

Italy can be viewed as a laboratory to assess the quality of mental healthcare delivered in a community-oriented system, especially for severe mental disorders, such as personality disorders. Although initiatives based on clinical indicators for assessing the quality of mental healthcare have been developed by transnational-organisations, there is still no widespread practice of measuring the quality of care pathways delivered to patients with severe mental disorders in a community-oriented system, especially using administrative healthcare databases. The aim of the study is to evaluate the quality of care delivered to patients with personality disorders taken-in-care by mental health services of four Italian regions (Lombardy, Emilia-Romagna, Lazio, Sicily).

**Methods:**

A set of thirty-three clinical indicators, concerning accessibility, appropriateness, continuity, and safety of care, was implemented using regional healthcare utilization databases, containing data on mental health treatments and diagnosis, hospital admissions, outpatient interventions and exams and drug prescriptions.

**RESULTS:**

31,688 prevalent patients with personality disorders treated in 2015 were identified, of whom 2,331 newly taken-in-care. One-in-10 patients received a standardized assessment, the treatment discontinuity affected half of the cases. 12.7% of prevalent patients received at least one hospitalization, 10.6% in the newly taken-in-care cohort. 6-out-of-10 patients had contact with community-services within 14 days from hospital discharge. Access to psychotherapy and psychoeducational treatments was low and delivered with a low intensity. The median of psychosocial interventions per person-year was 19.1 and 9.4, respectively, in prevalent and newly taken-in-care cases. Nearly 50% of patients received pharmacological treatments.

**Conclusions:**

Healthcare utilization databases were used to systematically evaluate and assess service delivery across regional mental health systems; suggesting that in Italy the public mental health services provide to individuals with personality disorders suboptimal treatment paths.

**Supplementary Information:**

The online version contains supplementary material available at 10.1186/s13033-023-00603-9.

## Background

Personality disorders, and in particular borderline personality disorder (BPD), constitute a real challenge for the mental health services system. Long neglected as diagnostic categories relevant to psychiatric nosography [1], their importance has been recognized for the increased treatment demand [2], for the strict relationship between self-harm and suicidality [3–5], for the evidence of associated high social and health costs [6, 7] and finally for the knowledge, acquired in recent decades, of offering effective treatments based on empirical evidence [8, 9], including the diffusion of Dialectical Behavioural Therapy [10].

In any case, it has been established that personality disorders are treatable and respond to manualized psychotherapies based on empowerment [11]. Unfortunately, in opposition to what is recommended by the guidelines [12, 13] the psychotherapies supplied by community mental health centers (CMHCs) are insufficient and furthermore there is a need for implementation models of the guidelines allowing local adaptation [11]. In the Italian mental health system, personality disorders, mainly BPD, constitute 14% of patients treated in community services and 20% of hospital admissions in psychiatric emergency wards [14], with a treated prevalence of 11 patients per 10,000 inhabitants [15]. Generally, patients with personality disorders receive predominantly pharmacological treatment and have less frequent access to adequate psychosocial treatments and psychotherapies [16, 17].

However, a further challenge is to ensure adequate quality of care for patients with severe mental disorders, especially for community-oriented system, like the Italian one. The quality of care must be measured, improved and communicated to all the stakeholders. Therefore, for the improvement of mental health services (MHS), it becomes crucial to implement a set of standardized and rigorous measures for a sound evaluation and monitoring process of the quality of care pathways delivered to patients with mental disorders. To address this issue, healthcare utilization (HCU) databases could represent a valuable source of complete, standardized, comparable, ready-to-use healthcare information, useful for the purpose of evaluation.

Given these premises, the Italian Ministry of Health (MoH) funded the multi-regional QUADIM project to assess the quality of “Clinical pathways for patients with severe mental disorders in Italy”. The present study, as part of the QUADIM project, represents the largest investigation of the quality of healthcare provided to patients with personality disorders engaged by Italian MHS. We used a set of indicators to assess the accessibility, timeliness, appropriateness, and safety of treatments currently provided, examining the strengths and weaknesses of MHS in four Italian regions (Lombardy, Emilia-Romagna, Lazio and Sicily).

## Methods

### Aim

The aim of the current study is to provide a tool to systematically evaluate and assess the quality of mental healthcare delivered to patients with personality disorders taken-in-care by Italian public services of mental health, using healthcare utilization databases.

### Setting

In Italy, in 1978 a reform law (e.g., “*Law Number 180*”) promoted the closure of public psychiatric hospitals and the implementation of a widespread and structured network of community mental health facilities, consolidating a community-based system of mental healthcare [18].

Thus, in Italy, the National health system (NHS) is decentralized and organized into public local health authorities, with each health authority having a department of mental health (DMH), which provides comprehensive mental healthcare to the target population. Each DMH manages a local network of community services (including CMHCs, general hospital psychiatric wards (GHPWs), day-care centers (DCs), and community residential facilities (CRFs)), which are required to provide at least the minimum level of services set by law. Private healthcare providers deliver day-care and residential care in conjunction with public DMHs.

### Data source

The data for this study were retrieved retrospectively from the HCU databases of four Italian regions (Lombardy, Emilia-Romagna, Lazio, and Sicily (restricted to the province of Palermo)). HCU data were available from the four regions for the 2013–2016 time interval at the beginning of the project, covering an overall adult resident population of 16 million people in the 2015 (according to the Italian Institute of Statistics, https://demo.istat.it/, last access on 28th August 2023).

All Italian citizens have equal access to healthcare as part of the NHS, and each region uses an automated system of HCU databases for the local management of the healthcare and its provision to residents. Indeed, HCU databases were originally established for recording all payments of healthcare providers to obtain reimbursement, thus storing, on ongoing basis, economic disease-related data from patients assisted by the Regional Health Service (i.e., a well-defined dynamic population). They include data on several services supplied to residents and collect a range of information, such as discharges from public or private hospitals, outpatient drug prescriptions, specialist visits and diagnostic exams, all reimbursable by the NHS. Furthermore, a national information system, specific for mental health, is also implemented by the regional DMHs and private facilities accredited by the NHS (the Italian “Mental Health Information System”, MHIS), collecting sociodemographic information, ICD-10 or ICD-9-CM diagnoses, and recording all treatments provided to all patients receiving mental healthcare. The entire list of interventions provided by community mental health services and recorded in the MHIS is reported in Supplementary **Table S1.** Data are registered and stored according to the Italian and European General Data Protection Regulation [19, 20].

Furthermore, since an anonymous identification code for each NHS beneficiary is recorded, it is possible to perform a record-linkage procedure which allows to interconnect HCU databases, enabling the study of the complete care pathway of NHS beneficiaries. Details of HCU databases use in mental health have been reported elsewhere [21–24].

### Harmonization and data processing

Although differences in the HCU databases across regions were limited, a between-region data harmonization was performed allowing the implementation of consistent and comparable data extraction processes (e.g., information of datasets and variables was uniformly encoded by using the same names, values and formats, etc.). Based on a detailed protocol describing data harmonization and extraction processes, regional anonymized data were extracted and processed locally by using common Statistical Analysis System (SAS) programs developed by two of the authors (Monzio Compagnoni and Caggiu). Diagnostic and therapeutic codes used are reported in Supplementary **Table S2**.

### Cohort selection

The target population consisted of all NHS beneficiaries residents in Lombardy, Emilia-Romagna, Lazio, and Sicily, aged 18–65 [22]. Those with a diagnosis of personality disorders who, from January to December 2015, had at least one contact with a DMH were identified. These patients were labelled as prevalent cases. The date of their first contact with a DMH during the recruitment was recorded as the index date. Then, to include the cohort of newly taken-in-care patients (e.g., those with first-lifetime diagnosis of personality disorders known to the NHS), prevalent cases were excluded if they (i) received a diagnosis of personality disorders at any time before the index date, (ii) experienced any hospital admission to a GHPW, and/or (iii) received at least two consecutive prescriptions for psychotropic drugs within the two years before the index date. Because there is some residual uncertainty regarding the ability of this algorithm to identify new diagnoses, the latter study cohort was restricted to patients aged 18–40 years [22, 24].

Members of both cohorts accumulated person-years of follow-up starting from the index date until one year after the index date (end of follow-up).

### Clinical indicators

Thirty-three quality indicators were jointly designed by two multidisciplinary expert groups, both funded by the Italian MoH (QUADIM-MAP projects, please see the Acknowledgements section) [22, 25], and they represent a general methodology suitable for the data research for personality disorders. Those indicators were designed starting from evidence-based recommendations tailored to community care goals produced with the agreement of the Italian MoH and regional governments [26], and considering the guideline developed by the National Institute for Clinical Excellence [27] as a milestone for the treatment of personality disorders. Recommendations, and the derived indicators, identified the interventions needed by essential clinical pathways for the treatment and monitoring of severe mental illnesses. Every indicator was analysed in accordance with different core dimensions of health quality (accessibility, continuity, appropriateness, and safety). A total of 33 clinical indicators were identified, each one related to a quality-dimension: accessibility and appropriateness (n = 23), continuity (n = 5) and safety (n = 5) of mental healthcare. More details on the rationale and process for identifying and constructing indicators to assess quality of care in severe mental disorders has been described elsewhere [23, 24, 28, 29].

### Statistical analysis

Prevalence and incidence rates, proportions and median values of the indicators were computed for each region and for the whole aggregated sample. As calculations were performed separately within each considered region, summarized estimates were obtained by pooling aggregated regional data.

The hypothesis of homogeneity among regional estimates was tested using (i) the chi-square test for clinical indicators expressed as proportions or (ii) the one-way analysis of variance (ANOVA) procedure for indicators expressed as the median number of interventions per person-years of follow-up [30]. Heterogeneity of estimates between regions was measured with the I^2^ statistics [31].

The prescriptions of drugs dispensed to patients during the follow-up were identified and used to evaluate persistence with the recommended pharmacotherapy. The duration of each prescription was calculated by the defined daily dose metric. Prescriptions were considered “consecutive” if the interval between the end of one prescription and the start of the following one was less than 90 days, and “interrupted” otherwise; interrupted prescriptions were considered to lead to discontinuation of treatment. All outpatient contacts provided by CMHCs or DCs were identified to evaluate the persistence with community care, and patients were considered persistent if they experienced at least one community contact every 90 days. The time spent in hospital and residential wards was considered continuity of care.

The standardized mortality ratio (SMR), which gives the ratio between observed and expected deaths, was calculated. The corresponding 95% CI were calculated by assuming that the observed number of deaths followed a Poisson distribution.

All the analyses were separately performed for each of the two considered cohorts and for each region, using the SAS Software (version 9.4; SAS Institute, Cary, NC, USA), and the R software (version 4.1.3, 2022, R Foundation for Statistical Computing, Vienna, Austria; packages: “*metamean*”, “*metamedian*”, “*readxl*”). For all hypotheses tested, two-tailed p-values less than 0.05 were considered significant.

## Results

**Fig. 1:**
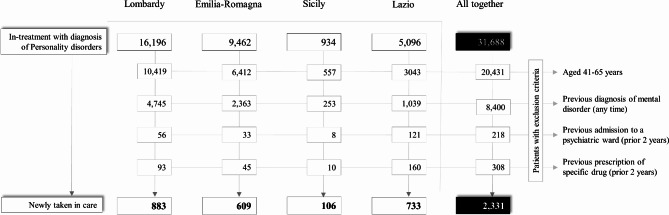
Flow-chart of inclusion and exclusion criteria for the eligibility of patients newly taken-in-care with personality disorders in three regions (Lombardy, Emilia-Romagna, Lazio) and one province (Palermo), and in the whole Italian sample. QUADIM-MAP Projects, Italy, 2015–2016

As shown in Fig. 1, patients with any diagnosis of personality disorders aged 18 or more treated by mental health services were 31,688, and constituted the prevalent cohort; cases newly taken-in-care during the follow up period of one year were 2,331. The age-, gender-standardized treated prevalent rates (per 10,000 inhabitants over the age of 18) were: Lombardy 19.5, Emilia-Romagna 25.3, Sicily 9.0, and Lazio 10.4. The overall prevalence rate was 17.6. In the newly taken-in-care cohort, the age-, gender-standardized rates (per 10,000 inhabitants aged between 18 and 40) are: 4.1 overall rate, 3.4 for Lombardy, 5.5 for Emilia-Romagna, 2.8 for Sicily and 4.7 for Lazio. The sociodemographic and diagnostic characteristics of the two study cohorts are shown in online Supplementary **Tables S3** and **S4**.

More than 9 out of 10 patients of the prevalent cohort had at least one contact (i.e., any kind of professional performance) with the CMHCs in the considered period; the number of newly taken-in-care cases that have had similar contacts is slightly lower. As for the intensity of contacts with the different types of professional, the median was 9.5 performances per person-year in the first cohort, 7.2 in the second. The discontinuity of territorial assistance concerned, respectively, almost half of the prevalent cohort and 67% of the newly taken-in-care cohort. In the prevalent cohort, 8 out of 10 patients received at least one psychiatric visit and, for treated subjects, the median number of visits per person-year was 4. In the newly taken-in-care cohort, the percentage of patients who received a psychiatric visit was 74.2%. Few prevalent patients received a standardized assessment; the numbers increase among the newly taken-in-care ones where, however, only one out of 10 patients of this group were measured with a psychometric method.

Overall, the psychosocial treatments, including professional performances directed to family members, psychoeducational interventions, and psychotherapies, were provided to more than half of the patients of both cohorts. Concerning psychotherapy, greater access was observed in patients newly taken-in-care (1 out of 3) compared to the prevalent cases (1 out of 5).

Concerning pharmacotherapies, almost half of prevalent patients were treated with antipsychotic agents, while only one newly taken-in-care patient out of four started a treatment with antipsychotics. Almost the same proportion was found regarding mood stabilizers drugs (45% and 29% for the prevalent and newly taken-in-care cohort, respectively), with valproic acid and carbamazepine being the most commonly dispensed stabilizing drugs.

Patients who experienced at least one hospitalization in a GHPW were 12.7% of the prevalent cohort and 10.6% of the newly taken-in-care one. Prevalent patients spent a median number of days of hospitalization in GHPW of two weeks, whereas for newly taken-in-care patients the median was nearly 10 days. Hospital readmissions in GHPW within 7 and 30 days, concerned about 15% and 30% of cases in both cohorts. Patients who received residential treatments were 15.7% and 11.1% for the prevalent and newly taken-in-care cohort, respectively. The median period of stay in a residential facility was 3 months for the prevalent cohort and 3.6 months for the newly taken-in-care one.

For both cohorts, the number of hospital discharges from a GHPW followed by at least an outpatient contact by CMHCs was approximately 60% within 14 days and 70% within 30 days. GHPW hospital discharges were rarely followed by home interventions within 14 days (4.1% and 1.9% for the two cohorts, respectively).

The discontinuity treatment with mood stabilizers occurred in 46.0% and 62.4% of patients on drug treatment, for the two cohorts, respectively.

The majority of patients under treatment with lithium in both cohorts (61.8% and 51.1%, respectively), received at least one blood control of lithium concentration and electrolytes. Among prevalent patients treated with antipsychotics, only one out of four received at least a complete set of recommended clinical controls within the year.

The standardized mortality rate was 2.06 (95% CI: 1.90–2.23) for the prevalent cohort and 1.70 (0.74–3.41) for newly taken-in-care patients. Both were higher than the mortality rate expected for the general population, although this excess mortality was not significant for newly taken-in-care patients.

Heterogeneity of the results among the regions was high. The overall outpatient interventions received at CMHCs in prevalent cohort were medially 9.5 per person-year, with a maximum of 13.0 (Emilia-Romagna) and a minimum of 7.0 (Sicily). For newly taken-in-care patients, the performance density was lower (7.2 performances per PY) with a wide variability between geographical areas. The overall discontinuity of the territorial care pathway in prevalent cohort was 39.8%: ranging from 31.2% (Lombardy) to 66.8% (Sicily). Half of the prevalent patients received at least one psychosocial intervention with a median 4.5 contacts per person-year, ranging from 3.0 (Sicily) to 6.0 (Lombardy).

## Discussion

Although data on services utilization by patients with personality disorder are affected by several factors and are difficult to compare [32], Italian mental health services guarantee accessibility to the care of these patients to an extent comparable to those of Netherlands, where the prevalence of BPD in patients treated by the CRFs is between 15 and 20% [33]. Similarly, in the United States, the prevalence of BPD patients is 10–12% in outpatient psychiatric settings and 20–22% in inpatient psychiatric settings [34]. The healthcare offer is fundamentally linked to the activity of local services, although there is a percentage of patients who, after a GHPW hospitalization, did not have further contact with the DMHs. The discrepancy between the two groups on this indicator suggests a greater difficulty in recruiting hospitalized patients not previously known by the community services. Data on timeliness showed that more than half of GHPW discharges were followed, for the two cohorts, by contact with the local facilities within the first 14 days. Newly taken-in-care patients exhibited a greater discontinuity of care. This data can be interpreted as a major criticality of the engagement phase in the construction of therapeutic relationship with patients at their first contact with an emergency setting.

Regarding the appropriateness it was found that prevalent patients adhere to long-term treatment more than newly taken-in-care patients. Major critical elements were highlighted through the analysis of other indicators. Indeed, it emerged that the majority of patients receive psychiatric treatment of a pharmacological type. Data on psychiatric visits, compared with those of psychosocial interventions, highlights the preponderance of medical treatments over psychotherapeutic, psychoeducational, and rehabilitative interventions. Only a minority of prevalent and newly taken-in-care patients received psychotherapy, with low intensity. Thus, access to psychotherapy is severely limited, while psychiatric intervention prevails in the treatment path. This data is consistent with what is reported in other works on the use of health resources by patients with personality disorder [35] showing that 61.5% of patients go to the medical psychiatrist and only 4.4% to the psychologist. Moreover, pharmacological treatments have extensively affected the population considered. One in 4 patients, in both cohorts, received interventions directed at family members, with a low intensity. Psychoeducational interventions also reached a minority component of the groups examined, with a negligible density of interventions. These results on appropriateness highlight that the *real care path* of patients with personality disorders, in contrast with the recommendations of the guidelines [12, 13], and even their adaptation to the national context [13], is strongly anchored in psychiatric medical treatment. A research found that 78% of patients with BPD take continuous pharmacological treatment and that 37% of these take a polypharmacy composed of three or more different molecules [36]. Other studies in European countries suggest that pharmacological treatment of personality disorders is largely privileged over psychotherapies [16] and confirm the problem of the use of polypharmacy, in the absence of a precise clinical rationale, to configure the risk of consequent iatrogenic effects [37]. Although evidence of efficacy has been collected for the use of mood stabilizers and low-dose antipsychotics for the treatment of some core symptoms of BPD [38], we are far from considering effective drug therapies in the overall treatment of the disorder [39–41]. Psychotherapeutic and psychosocial interventions, widely recommended as the first line of treatment [8, 11], are intended for a minority component and with a frequency of interventions below acceptable standards. The use of residential interventions is very relevant for both the percentage of patients involved and the length of stay in the facilities. This element also contradicts the indications of the recommendations that negatively consider long-term residential programs for patients with personality disorders [12] and with studies carried out in England that hypothesize the iatrogenicity of long-term residential treatments for patients with BPD [42]. The use of hospitalization must be considered along the same line, suggesting that access to GHPW is dominant in the treatment of crises, in contrast to indications based on the development of individual capability to increase coping skills [43]. The offer of hospitalization for patients with BPD has been criticized for its ineffectiveness in preventing the risk of suicide and for the potential negative effects of this strategy [44] in comparison with intensive outpatient treatments [45].

The performance of services related to the safety of care, provides an uneven picture. Indeed, the number of patients who received at least one control of lithaemia (among patients treated with lithium) is satisfactory, even if it can be improved. On the other hand, the indicator relating to glycaemic controls shows that only a minority of cases treated with antipsychotics receive at least one set of recommended clinical controls.

Finally, study findings were obtained by the implementation of a set of indicators representing different clinical domains of care pathways delivered by mental health services. Since data were retrieved from the current administrative healthcare databases, it was possible to identify a large and unselected cohort of patients with personality disorders taken-in-care by Italian public services, which has no terms of comparison with other surveys conducted in the European countries. Studies that have evaluated the frequency of borderline disorder in clinical settings are on far fewer series [34]. The QUADIM project, made it possible to analyse healthcare pathways delivered to patients suffering from personality disorders under multiple quality dimensions and aspects, such as contact events, drug therapies and hospital-territory continuity. The QUADIM project, also promoted the definition and implementation of standardized and routine measures for the monitoring, evaluation and comparison of the complete care pathway delivered to patients. In other words, it was provided a methodology which use administrative healthcare data to systematically assess service delivery across the (regional) mental health systems. Indeed, using indicators to systematically evaluate health service delivery allows to identify critical issues and provide useful information for improving the treatment process; investigating effectiveness and safety of health services provided by an NHS.

To better understand the results obtained, it is necessary to examine the study strengths and limitations. In Italy, healthcare is both free and universal, allowing all citizens to access essential levels of healthcare free of charge. As a result, this survey was based on data from a large, unselected population. Accordingly, the present study can be reasonably defined as “population-based”, offering guarantees of representativeness and generalizability, since we were able to include all beneficiaries who were treated by public services for a given condition. The availability of high-quality integrated individual data, allowed to assess the complete care pathway of patients with personality disorders, in a context which reflects current clinical practice, generating reliable evidence. Indeed, with the use of HCU databases as data source, it was possible to link data from public and private generic healthcare with those on outpatient and inpatient mental healthcare. Information on psychiatric care delivered were retrieved from the Italian MHIS, which is an information system regulated by law at national level. By means of the MHIS, we have access to a unique and complete data source, allowing comparisons among regions. Also, newly taken-in-care patients were identified at the time of their first contact with NHS mental health services, and their full mental healthcare pathway was recorded from their first diagnoses with a personality disorder.

However, from the use of HCU databases also derived some limitations. The first is constituted by the heterogeneity of the diagnostic spectrum of personality disorders. Most of the clinical studies concern BPD and comparisons with literature data have been part of the studies carried out mainly with this specific diagnostic category. Moreover, very often in clinical practice the personality disorder, generally understood, refers to clinical pictures with common characteristics with the BPD. Personality disorder diagnoses have only rarely been substantiated using standardized diagnostics. There is another typical problem of HCU databases, namely the lack of information on the clinical severity and comorbidities, well known modifiers of treatment outcome and patients’ adherence. Furthermore, there are some indicators of quality of care that cannot be assessed because of the lack of information in the MHIS. However, our work provides detailed information on the quality of care received by people with personality disorders in Italian public mental health services. The areas investigated with the system of indicators were accessibility, appropriateness, continuity, safety of care and geographic variability. Regarding European literature, just few works have previously investigated that topic analysing the use of mental health services by patients with personality disorders [46] or the type of professionals consulted in a very select sample of users [35]. Only the US National Comorbidity Survey study [47], provides data on the use of health services by a very large sample of patients with BPD [48]. But of course, the different organization of health services and the epidemiological differences between European countries and United States [49], make the results less comparable. However, we cannot completely rule out that some differences between regions may occur because of the heterogeneity in data quality and completeness. When the study and the data management of the HCU used started the most recent databases available were related to the years 2013–2016. Some differences may have occurred since 2015 and, but not of such an extent to modify or invalidate our findings.

Furthermore, this study examines the demand for healthcare treatment from patients with borderline disorder but does not address the issue of the quality of the care offered. Finally, the validity of some estimates assumes that the prescription of a drug or the provision of a service corresponds to the consumption of the drug or the execution of the clinical control. Nonetheless, there is no guarantee that this will always be the case, and it is quite likely that prescriptions do not always result in drug consumption.

## Conclusions

This investigation conducted using the indicators obtained from the National MHIS and other administrative health archives, allows us to state that personality disorders are widely represented in the population treated by the DMHs, by means of a structured and easy-to-implement tool for the assessment and monitoring of healthcare provided. The implementation of standardized measures allows to systematically and routinely evaluate the quality of psychiatric care regularly provided to patients with personality disorders taken-in-care by Italian public services of mental health services.

From the elements collected with our research, mental health services need to update significantly the professional culture and to equip themselves to expand the offer of psychosocial treatments, in particular individual and group psychotherapies. The wide variability found among the centres leads us to believe that the quality of services offered presents important differences which are due to both structural and also occasional factors, such as the training and functional specialization of the professionals (given the lack of professionals specialized in treatment of personality disorders), and the willingness of the management of healthcare to invest resources. The implementation of recommended guidelines is hampered by similar impediments, both in Italy as in other European countries [50]. Moreover, access to evidence-based therapies is also severely limited by economic and organizational factors [16]. A possible solution to address these problems is the dissemination of regional programs capable of satisfying the training need of clinicians and facilitating the implementation of intervention models based effective therapies [14, 50]. The implementation of the common factors of effective therapies, the structuring of the treatment path and the enhancement of the basic skills of professionals, could be the best way to improve the quality of care offered to patients with personality disorders in public mental health services. Those system offers the opportunity to trace and evaluate the complete care pathway of patients with severe mental disorders, in a setting reflecting current clinical practice. Thereby, generating reliable real-world evidence on mental healthcare could be useful for guide the implementation of specific health policies.

In conclusion, with this paper it was shown that a set of clinical indicators, retrieved from HCU database, could be a useful tool for monitoring the quality of health care in a mental health system in an automated and standardized way. Beyond their function for the monitoring and the assessment, they could also be useful to (i) make benchmarking among countries/regions, (ii) establish critical issues and priorities for intervention and quality improvement and (iii) support accountability in mental health care.

**Table 1:** Clinical indicators estimated, in the first year of follow-up, for prevalent patients with personality disorders treated by local DMHs, stratified for area (Lombardy, Emilia Romagna and Lazio Regions and Province of Palermo) and in the whole sample. QUADIM-MAP projects, Italy, 2015–2016

		Lombardia (n = 16,196)		Emilia-Romagna (n = 9,462)		Palermo (n = 934)		Lazio (n = 5,096)		Whole sample (n = 31,688)		I^2^ ¥			
	Age-, gender-standardized treated prevalence rate (x 10,000)	19.5		25.3		9.0		10.4		**17.6**					
**ACCESSIBILITY AND APPROPRIATENESS OF MENTAL HEALTH CARE**															
1	Patients with at least one outpatient contact in CMHCs or DCs	92.3%		98.4%		74.2%		97.4%		**94.4%**		99			
2	Median number of outpatient contacts in CMHCs *(per PY)*	8.0		13.0		7.0		10.0		**9.5**		99			
3	Patients with at least one psychiatric visit	81.7%		83.9%		65.1%		76.5%		**81.0%**		99			
4	Median number of outpatient psychiatric visits *(per PY)*	4.0		4.0		3.0		5.0		**4.0**		99			
5	Patients with at least one standardized assessment using tests	4.3%		2.6%		2.2%		5.0%		**3.8%**		97			
6	Median number of standardized assessments using tests *(per PY)*	1.0		1.0		1.0		1.0		**1.0**		0			
7	Patients treated with at least one psychosocial intervention in CMHCs	52.4%		51.5%		52.6%		59.0%		**53.2%**		97			
8	Median number of psychosocial interventions in CMHCs *(per PY)*	6.0		4.0		3.0		5.0		**4.5**		98			
9	Patients treated with at least one psychoeducation session **‡**	3.1%		4.9%		6.2%				**3.8**%		97			
10	Median number of psychoeducation sessions *(per PY)***‡**	3.0		2.8		1.0				**2.6**		74			
11	Patients treated with at least one psychotherapy session	20.7%		9.5%		19.7%		32.4%		**19.2**%		99			
12	Median number of psychotherapy sessions *(per PY)*	6.0		5.0		3.5		8.0		**5.7**		98			
13	Median number of interventions specifically addressed to patients’ family members *(per PY)*	2.0		2.0		2.0		2.0		**2.0**		0			
14	Patients treated with antipsychotic agents	48.7%		56.6%		32.8%		39.2%		**49.1**%		99			
15	Patients treated with Mood Stabilizers	44.1%		50.2%		37.8%		40.5%		**45.1%**		98			
	*Patients treated with Lithium*	3.8%		3.9%		1.8%		3.6%		**3.8**%		85			
	*Patients treated with Valproic acid, Carbamazepine*	16.8%		18.0%		26.3%		22.8%		**18.4**%		97			
16	Patients treated with Antidepressant agents	46.1%		55.4%		32.0%		31.7%		**46.1%**		99			
17	Patients with at least one admission in residential facilities	17.5%		16.8%		2.0%		10.2%		**15.7%**		99			
18	Median number of days spent in residential facilities *(per PY)*	33.0		46.0		261.2		78.1		**95.7**		99			
19	Patients with at least one admission in GHPW	14.2%		12.4%		12.2%		8.6%		**12.7**%		98			
20	Median number of days spent in GHPW *(per PY)*	14.0		13.5		18.0		15.0		**14.0**		0			
21	Admissions with a length of stay in GHPW higher than 30 days	5.8%		2.3%		5.5%		4.7%		**5.0**%		92			
22	Unplanned re-admissions in GHPW within 7 days^**¶**^	14.1%		17.6%		16.1%		15.3%		**15.0**%		65			
23	Unplanned re-admissions in GHPW within 30 days^**¶**^	27.3%		31.6%		35.5%		27.8%		**28.5**%		77			
**CONTINUITY OF MENTAL HEALTH CARE**															
24	Patients with continuous community care	68.8%		58.4%		33.2%		47.1%		**61.2%**		99			
25	Patients persistent with Mood stabilizers therapy	49.6%		59.4%		56.1%		56.1%		**54.0%**		97			
26	GHPW discharges followed by any mental health outpatient contact within 14 days	54.4%		77.0%		51.6%		67.0%		**60.4%**		99			
27	GHPW discharges followed by an outpatient psychiatric visit within 14 days	34.8%		41.9%		40.1%		46.3%		**37.8%**		93			
28	GHPW discharges followed by home care within 14 days **§**	4.4%				1.4%		3.4%		**4.1%**		84			
**SAFETY OF MENTAL HEALTH CARE**															
29	Patients monitored for hyperglycaemia and hyperlipidaemia *(in patients treated with antipsychotics)*	26.4%		26.4%		25.5%		20.3%		**25.6**%		92			
30	Patients monitored with Lithaemia *(in patients treated with Lithium)*	69.1%		57.9%		41.2%		47.8%		**61.8**%		91			
31	Patients with a complete set of clinical controls *(in patients treated with Valproic acid, Carbamazepine)*	53.9%		44.8%		37.0%		40.1%		**47.8**%		97			
32	Patients with a complete set of clinical controls *(in patients treated with Lamotrigine)*	46.4%		38.8%		30.8%		37.0%		**41.5**%		56			
33	Mortality (SMR), and relative 95% CI	2.19 (1.96 to 2.45)		2.08 (1.82 to 2.37)		2.59 (1.69 to 3.84)		1.46 (1.12 to 1.87)		**2.06** **(1.90 to 2.23)**					

DMH: department of mental health. CMHC: community mental health centres; DC: day-care centres; PY: person-year; FGAs: first generation antipsychotics; SGAs: second generation antipsychotics; GHPW: general hospital psychiatric wards; SMR: standardized mortality ratio

* P-value < 0.05 for test of homogeneity among indicators’ regional estimates

**§** Information for Emilia-Romagna Region was not available for this clinical indicator, which was calculated on the 22,226 remaining patients

**ψ** Psychosocial interventions are intended excluding psychotherapy and psychoeducation sessions

**‡** Information for Lazio Region was not available for this clinical indicator, which was calculated on the 26,592 remaining patients

**¶** After a previous hospital admission in GHPW (statistical unit)

**¥** Values of I^2^ for heterogeneity are percentages and can be classified in: Negligible (0–25); Moderate (26–50); Substantive (51–75); Considerable (76–100)

**Table 2:** Clinical indicators estimated, in the first year of follow-up, for patients newly taken-in-care with personality disorders treated by local DMHs, stratified for area (Lombardy, Emilia Romagna and Lazio Regions and Province of Palermo) and in the whole sample. QUADIM-MAP projects, Italy, 2015–2016

		Lombardia (n = 883)	Emilia-Romagna (n = 609)	Palermo (n = 106)	Lazio (n = 733)	Whole sample (n = 2,331)	I^2^ ¥
	Age-, gender-standardized treated prevalence rate (x 10,000)	3.4	5.5	2.8	4.7	**4.1**	
**ACCESSIBILITY AND APPROPRIATENESS OF MENTAL HEALTH CARE**							
1	Patients with at least one outpatient contact in CMHCs or DCs	82.0%	96.7%	84.0%	98.0%	**90.9%**	98
2	Median number of outpatient contacts in CMHCs *(per PY)*	8.0	8.0	7.0	6.0	**7.2**	73
3	Patients with at least one psychiatric visit	70.2%	85.9%	71.7%	69.6%	**74.2%**	97
4	Median number of outpatient psychiatric visits *(per PY)*	4.0	4.0	3.0	3.0	**3.6**	81
5	Patients with at least one standardized assessment using tests	12.2%	7.7%	9.4%	11.3%	**10.6%**	98
6	Median number of standardized assessments using tests *(per PY)*	1.0	2.0	1.0	1.0	**1.0**	0
7	Patients treated with at least one psychosocial intervention in CMHCs	53.9%	50.2%	65.1%	58.5%	**54.9**%	83
8	Median number of psychosocial interventions in CMHCs *(per PY)*	5.0	3.0	4.0	4.0	**4.0**	71
9	Patients treated with at least one psychoeducation session **‡**	3.4%	3.8%	4.7%		**3.6**%	0
10	Median number of psychoeducation sessions *(per PY)***‡**	3.0	3.0	1.0		**2.9**	0
11	Patients treated with at least one psychotherapy session	37.9%	19.9%	37.7%	40.5%	**34.0**%	99
12	Median number of psychotherapy sessions *(per PY)*	5.0	5.0	3.5	6.0	**5.1**	14
13	Median number of interventions specifically addressed to patients’ family members *(per PY)*	2.0	2.0	2.0	1.0	**1.7**	83
14	Patients treated with antipsychotic agents	27.2%	34.2%	21.7%	21.0%	**26.8%**	90
15	Patients treated with Mood Stabilizers	30.2%	36.0%	31.1%	21.4%	**29.0%**	92
	*Patients treated with Lithium*	1.9%	3.4%	1.9%	1.0%	**2.0%**	70
	*Patients treated with Valproic acid, Carbamazepine*	13.5%	13.1%	24.5%	12.3%	**13.5%**	62
16	Patients treated with Antidepressant agents	38.4%	43.7%	21.7%	22.8%	**34.1%**	97
17	Patients with at least one admission in residential facilities	13.1%	13.1%	0.9%	8.3%	**11.1%**	97
18	Median number of days spent in residential facilities *(per PY)*	15.0	27.0	365.2	51.0	**110.7**	99
19	Patients with at least one admission in GHPW	12.6%	13.6%	10.4%	5.6%	**10.6**%	92
20	Median number of days spent in GHPW *(per PY)*	14.0	12.0	25.0	11.0	**12.8**	0
21	Admissions with a length of stay in GHPW higher than 30 days	3.5%	5.3%	10.0%	1.7%	**4.2%**	92
22	Unplanned re-admissions in GHPW within 7 days^**¶**^	9.9%	18.4%	30.0%	13.3%	**14.2%**	60
23	Unplanned re-admissions in GHPW within 30 days^**¶**^	20.5%	26.3%	50.0%	20.0%	**24.3%**	71
**CONTINUITY OF MENTAL HEALTH CARE**							
24	Patients with continuous community care	51.2%	23.4%	20.2%	25.2%	**33.4%**	99
25	Patients persistent with Mood stabilizers therapy	34.5%	40.6%	39.4%	38.2%	**37.6%**	0
26	GHPW discharges followed by any mental health outpatient contact within 14 days	61.4%	72.4%	53.3%	48.3%	**60.8%**	68
27	GHPW discharges followed by an outpatient psychiatric visit within 14 days	40.4%	56.6%	33.3%	30.0%	**41.5%**	75
28	GHPW discharges followed by home care within 14 days **§**	1.8%		3.3%	1.7%	**1.9%**	0
**SAFETY OF MENTAL HEALTH CARE**							
29	Patients monitored for hyperglycaemia and hyperlipidaemia *(in patients treated with antipsychotics)*	12.1%	13.0%	13.0%	9.7%	**11.8**%	0
30	Patients monitored with lithaemia *(in patients treated with Lithium)*	58.8%	47.6%	50.0%	42.9%	**51.1**%	0
31	Patients with a complete set of clinical controls *(in patients treated with Valproic acid, Carbamazepine)*	46.2%	38.8%	34.6%	21.1%	**36.2**%	82
32	Patients with a complete set of clinical controls *(in patients treated with Lamotrigine)*	54.5%	18.8%	0%	12.5%	**27.0**%	25
33	Mortality (SMR), and relative 95% CI	2.47 (0.24 to 9.63)	6.56 (1.72 to 17.5)	0	0.69 (0.10 to 2.69)	**1.70** **(0.74 to 3.41)**	

DMH: department of mental health. CMHC: community mental health centres; DC: day-care centres; PY: person-year; FGAs: first generation antipsychotics; SGAs: second generation antipsychotics; GHPW: general hospital psychiatric wards; SMR: standardized mortality ratio

* P-value < 0.05 for test of homogeneity among indicators’ regional estimates

**§** Information for Emilia-Romagna Region was not available for this clinical indicator, which was calculated on the 1,722 remaining patients

**ψ** Psychosocial interventions are intended excluding psychotherapy and psychoeducation sessions

**‡** Information for Lazio Region was not available for this clinical indicator, which was calculated on the 1,598 remaining patients

**¶** After a previous hospital admission in GHPW (statistical unit)

**¥** Values of I^2^ for heterogeneity are percentages and can be classified in: Negligible (0–25); Moderate (26–50); Substantive (51–75); Considerable (76–100)

### Electronic supplementary material

Below is the link to the electronic supplementary material.

Supplementary Material 1

## Data Availability

The data that support the findings of this study are available from the Regions of Lombardy, Lazio and Emilia-Romagna, and the Province of Palermo, but restrictions apply to the availability of these data, which were used under license for the current study, and so are not publicly available. Data are however available from the authors upon reasonable request and with permission of the Regions involved in this study.

## Abbreviations

CMHCs: Community Mental Health Centers
BPD: Borderline Personality Disorder
DMHs: Departments of Mental Health
NHS: National Health Service
HCU: Healthcare Utilization databases
GHPWs: General Hospital Psychiatric Wards
DCs: Day-Care Centers
CRFs: Community Residential Facilities
MHIS: Mental Health Information System
ICD: International Classification of Diseases
ATC: Anatomical Therapeutic Chemical Classification System
SAS: Statistical Analysis System software
MoH: Ministry of Health
QUADIM: “Clinical pathways in patients with severe mental disorders in Italy” project
SMR: Standardized Mortality Ratio
ANOVA: Analysis of Variance
