# Supplementary material for: Assessing the quality of the care offer for people with personality disorders in Italy: the QUADIM project. A multicentre research based on the database of use of Mental Health services
Source: Int J Ment Health Syst. 2023 Oct 13;17:31. doi: 10.1186/s13033-023-00603-9 (PMC10571410; doi:10.1186/s13033-023-00603-9)
Supplement: Supplementary file 1 — Supplementary Material 1 [file 13033_2023_603_MOESM1_ESM.docx]

**Assessing the quality of the care offer for people with personality disorders in Italy: the QUADIM project. A multicentre research based on the database of use of Mental Health services.**

Michele SANZA ^1^, Matteo MONZIO COMPAGNONI ^2,3^, ORCID Id: [0000-0002-2105-4572](https://orcid.org/0000-0002-2105-4572), Giulia CAGGIU ^2,3,4^, ORCID Id: [0000-0002-6631-5393](https://orcid.org/0000-0002-6631-5393), Liliana ALLEVI ^4^, Angelo BARBATO ^5^, ORCID Id: [0000-0001-7142-700X](https://orcid.org/0000-0001-7142-700X), Jeannette CAMPA ^6^, Flavia CARLE ^3,7^, ORCID Id: [0000-0002-2422-6528](https://orcid.org/0000-0002-2422-6528), Barbara D'AVANZO ^5^, ORCID Id: [0000-0002-0361-7263](https://orcid.org/0000-0002-0361-7263), Teresa DI FIANDRA ^8^, ORCID Id: [0000-0003-1124-0600](https://orcid.org/0000-0003-1124-0600), Lucia FERRARA ^9^, ORCID Id: [0000-0001-6955-8983](https://orcid.org/0000-0001-6955-8983), Andrea GADDINI ^10^, Alessio SAPONARO ^11^, Salvatore SCONDOTTO ^3, 12^, ORCID Id: [0000-0001-7620-3080](https://orcid.org/0000-0001-7620-3080), Valeria D TOZZI ^9^, ORCID Id: [0000-0002-0660-7853](https://orcid.org/0000-0002-0660-7853), Stefano LORUSSO^13^, Cristina GIORDANI^13^, Giovanni CORRAO ^2,3^, ORCID Id: [0000-0002-1034-8444](https://orcid.org/0000-0002-1034-8444), Antonio LORA ^3,4^, ORCID Id: [0000-0002-6806-5199](https://orcid.org/0000-0002-6806-5199)

^1^ Department of Mental Health and Addiction Disorders Forlì-Cesena, AUSL Romagna, Cesena, Italy

^2^ Unit of Biostatistics, Epidemiology and Public Health, Department of Statistics and Quantitative Methods, University of Milano-Bicocca, Milan, Italy

^3^ National Centre for Healthcare Research and Pharmacoepidemiology, University of Milano-Bicocca, Milan, Italy

^4^ Department of Mental Health and Addiction Services, ASST Lecco, Lecco, Italy

^5^ Department of Health Policy, Istituto di Ricerche Farmacologiche Mario Negri IRCCS, Milano, Italy

^6^ Addiction Unit, AUSL Romagna, Cesena, Italy

^7^ Center of Epidemiology and Biostatistics, Polytechnic University of Marche, Ancona, Italy

^8^ Psychologist, previously General Directorate for Health Prevention, Ministry of Health, Rome, Italy

^9^ Centre of Research on Health and Social Care Management, CERGAS SDA Bocconi School of Management (Bocconi University) Milan, Italy

^10^ Agency for Public Health, Lazio Region, Rome, Italy

^11^ General Directorate of Health and Social Policies, Emilia-Romagna Region, Bologna, Italy

^12^ Department of Health Services and Epidemiological Observatory, Regional Health Authority, Sicily Region, Palermo, Italy

^13^ Department of Health Planning, Italian Health Ministry, Rome, Italy

**SUPPLEMENTARY MATERIAL**

**Address for correspondence**: Dr. Matteo Monzio Compagnoni, PhD; Division of Biostatistics, Epidemiology and Public Health, Department of Statistics and Quantitative Methods, University of Milano-Bicocca, Street Bicocca degli Arcimboldi, 8, Building U7, 20126 Milan, Italy.
E-mail: [matteo.monziocompagnoni@unimib.it](mailto:matteo.monziocompagnoni@unimib.it)

**Supplementary Table S1.** Service interventions, treatments and activities delivered by community mental health centers (CMHCs) and day centers (DCs), and their classification in the Italian Mental Health Information System.

| **Interventions and activities** | **Italian Mental Health Information system codes** |
| --- | --- |
|  |  |
| **Generic care** |  |
| Psychiatric visit | 01 |
| Individual meeting with a professional | 03 |
| Consultation | 04 |
| Medico-legal assessment | 05 |
| Psychological testing | 06 |
| Drug administration | 11 |
| Meeting with relatives | 12 |
| Staff meeting | 15 |
| Attendance to day centre | 20 |
| Support to daily living activity | 24 |
| Network interventions | 26 |
| **Psychosocial interventions** |  |
| Individual living skills training | 16 |
| Group living skills training | 17 |
| Individual socialization | 18 |
| Socialization group | 19 |
| Expressive, manual and bodywork individual interventions | 21 |
| Expressive, manual and bodywork group interventions | 22 |
| Vocational training | 23 |
| Support for financial, welfare procedures and leisure activities | 25 |
| **Psychotherapy** |  |
| Psychological visit | 02 |
| Individual psychotherapy | 07 |
| Couple psychotherapy | 08 |
| Family psychotherapy | 09 |
| Group psychotherapy | 10 |
| **Psychoeducation** |  |
| Single family psychoeducation | 13 |
| Multifamily group psychoeducation | 14 |

**Supplementary Table S2.** Diagnostic and therapeutic (ICD-9-CM, ICD-10, and ATC) codes used in the current study for drawing records and fields from healthcare utilization databases.

| **PERSONALITY DISORDER** | |
| --- | --- |
|  | **ICD-10 codes**  **(Lombardy)** |
| Specific personality disorders | F60.* |
| Mixed and other personality disorders | F61.* |
|  | **ICD-9-CM codes**  **(Emilia-Romagna, Lazio and Palermo)** |
| Personality disorders | 301.* |
|  | **ATC codes** |
| Lithium | N05AN |
| Lamotrigine | N03AX09 |
| Valproic acid, Carbamazepine | N03AG01, N03AF01 |
| Second generation antipsychotics (SGAs) |  |
| Olanzapine | N05AH03 |
| Quetiapine | N05AH04 |
| Aripiprazole | N05AX12 |
| **OUTPATIENT PROCEDURES** | |
|  | **National procedure codes** |
| Glycated haemoglobin | 90.27.1, 90.28.1 |
| Lipid profile | 90.14.1, 90.14.3, 90.43.2 |
| Electrolytes | 91492, 90404, 90374, 90133 |
| Lithaemia | 90322 |
| Complete blood count | 90622 |
| Liver function | 90045, 90092, 90255 |
| Psychiatric visit | 94.12.1, 94.19.1 |
| Psychological interview | 94.09 |
| Standardized assessments using tests | 94.01.1, 94.08.3, 94.08.4, 94.08.5, 94.08.6 |
| Couple/family psychotherapy | 94.3, 94.42 |
| Group psychotherapy | 94.44 |

**Supplementary Table S3.** Baseline characteristics of prevalent patients with personality disorder treated by DMHs of four Italian areas (Lombardy, Emilia Romagna and Lazio Regions and Province of Palermo) and in the whole sample. Italy, QUADIM-MAP projects, Italy, 2015-2016.

|  | **Lombardy**  (N=16,196) | **Emilia-Romagna**  (N=9,462) | **Palermo**  (N=934) | **Lazio**  (N=5,096) | **Whole sample**  (N=31,688) |
| --- | --- | --- | --- | --- | --- |
|  |  |  |  |  |  |
| **Gender** |  |  |  |  |  |
| Men | 8,243 (50.9%) | 4,374 (46.2%) | 535 (57.3%) | 2494 (48.9%) | 15,646 (49.4%) |
| **Age (years)** |  |  |  |  |  |
| Mean (SD) | 45.2 (13.4) | 46.8 (14.0) | 44.5 (15.0) | 43.9 (13.9) | 45.1 (14.1) |
| 18-25 | 2,476 (15.3%) | 708 (7.5%) | 112 (12.0%) | 600 (11.8%) | 3,896 (12.3%) |
| 26-40 | 3,301 (20.4%) | 2,342 (24.8%) | 265 (28.4%) | 1,453 (28.5%) | 7,361 (23.2%) |
| 41-49 | 4,426 (27.3%) | 2,492 (26.3%) | 214 (22.9%) | 1,256 (24.6%) | 8,388 (26.5%) |
| ≥50 | 5,993 (37.0%) | 3,920 (41.4%) | 343 (36.7%) | 1,787 (35.1%) | 12,043 (38.0%) |
| **Education years** |  |  |  |  |  |
| 0-5 | 4,536 (28.0%) | 1,046 (11.1%) | 207 (22.2%) | 428 (8.4%) | 6,217 (19.7%) |
| 6-8 | 8,075 (49.9%) | 3,956 (41.8%) | 669 (71.6%) | 2,074 (40.7%) | 14,774 (46.6%) |
| 9-13 | 2,216 (13.7%) | 3,037 (32.1%) | 0 (0%) | 1,789 (35.1%) | 7,042 (22.2%) |
| ≥14 | 430 (2.7%) | 608 (6.4%) | 57 (6.1%) | 406 (8.0%) | 1,501 (4.7%) |
| *Missing data* | *939 (5.8%)* | *815 (8.6%)* | *1 (0.1%)* | *399 (7.8%)* | *2,154 (6.8%)* |
| **Job condition** |  |  |  |  |  |
| Employed | 8,310 (51.3%) | 2,681 (28.3%) | 168 (18.0%) | 1,747 (34.3%) | 12,906 (40.7%) |
| Unemployed | 4,402 (27.2%) | 4,376 (46.2%) | 736 (78.8%) | 2,981 (58.5%) | 12,495 (39.4%) |
| Invalid | 2,646 (16.3%) | 440 (4.7%) | 28 (3.0%) | 1 (0.0%) | 3,115 (9.9%) |
| *Missing data* | *838 (5.3%)* | *1,965 (20.8%)* | *2 (0.2%)* | *367 (7.2%)* | *3,172 (10.0%)* |
| **Family arrangement ^§^** |  |  |  |  |  |
| Living with family | 12,037 (74.3%) | 6,509 (68.8%) | 230 (24.6%) | NA | 18,776 (70.6%) |
| Living in community | 861 (5.3%) | 268 (2.8%) | 87 (9.3%) | NA | 1,216 (4.6%) |
| Living alone | 2,625 (16.2%) | 1,592 (16.8%) | 52 (5.6%) | NA | 4,269 (16.0%) |
| *Missing data* | *673 (4.2%)* | *1,093 (11.6%)* | *565 (60.5%)* | *NA* | *2,331 (8.8%)* |
| **Marital status** |  |  |  |  |  |
| Unmarried | 9,376 (57.9%) | 5,008 (52.9%) | 456 (48.8%) | 3,064 (60.1%) | 17,904 (56.5%) |
| Married | 3,874 (23.9%) | 2,224 (23.5%) | 326 (34.9%) | 1,072 (21.0%) | 7,496 (23.7%) |
| Separated | 1,170 (7.2%) | 630 (6.6%) | 64 (6.9%) | 367 (7.2%) | 2,231 (7.1%) |
| Divorced | 924 (5.7%) | 659 (7.0%) | 22 (2.4%) | 241 (4.7%) | 1,846 (5.8%) |
| Widow/er | 294 (1.8%) | 236 (2.5%) | 31 (3.3%) | 60 (1.2%) | 621 (1.9%) |
| *Missing data* | *558 (3.4%)* | *705 (7.5%)* | *35 (3.7%)* | *292 (5.8%)* | *1,590 (5.0%)* |
| **Clinical status†** |  |  |  |  |  |
| Optimal | 6,240 (38.5%) | 4,104 (43.4%) | 386 (41.3%) | 2,300 (45.1%) | 13,030 (41.1%) |
| Good | 3,459 (21.4%) | 2,621 (27.7%) | 202 (21.6%) | 1,096 (21.5%) | 7,378 (23.3%) |
| Intermediate | 4,406 (27.2%) | 1,653 (17.5%) | 225 (24.1%) | 1,131 (22.2%) | 7,415 (23.4%) |
| High-intermediate | 1,196 (7.4%) | 550 (5.8%) | 76 (8.2%) | 319 (6.3%) | 2,141 (6.8%) |
| Poor | 895 (5.5%) | 534 (5.6%) | 45 (4.8%) | 250 (4.9%) | 1,724 (5.4%) |

§ Information for Lazio Region was not available for this characteristic, which was calculated on the 26,592 remaining patients.

† The clinical status was assessed by the Multisource Comorbidity Score (MCS) according to the hospital admission and the drugs prescribed in the two-year period before the index date. Five categories of clinical status were considered: optimal (score=0), good (1≤score≤5), intermediate (6≤score≤10), high-intermediate (11≤score≤15) and poor (score ≥ 16).

**Supplementary Table S4.** Baseline characteristics of patients newly engaged in services with personality disorder treated by DMHs of four Italian areas (Lombardy, Emilia Romagna and Lazio Regions and Province of Palermo) and in the whole sample. Italy, QUADIM-MAP projects, Italy, 2015-2016.

|  | **Lombardy**  (N=883) | **Emilia-Romagna**  (N=609) | **Palermo**  (N=106) | **Lazio**  (N=733) | **Whole sample**  (N=2,331) |
| --- | --- | --- | --- | --- | --- |
|  |  |  |  |  |  |
| **Gender** |  |  |  |  |  |
| Men | 434 (49.2%) | 274 (45.0%) | 66 (62.3%) | 379 (51.7%) | 1,153 (49.5%) |
| **Age (years)** |  |  |  |  |  |
| Mean (SD) | 27.7 (7.0) | 28.8 (7.0) | 28.7 (7.6) | 29.7 (7.0) | 28.7 (7.1) |
| 18-25 | 545 (61.7%) | 350 (57.5%) | 62 (58.5%) | 387 (52.8%) | 1,344 (57.7%) |
| 26-40 | 338 (38.3%) | 259 (42.5%) | 44 (41.5%) | 346 (47.2%) | 987 (42.3%) |
| **Education years** |  |  |  |  |  |
| 0-5 | 186 (21.1%) | 13 (2.1%) | 7 (6.6%) | 36 (4.9%) | 242 (10.4%) |
| 6-8 | 425 (48.1%) | 201 (33.0%) | 92 (86.8%) | 313 (42.7%) | 1,031 (44.2%) |
| 9-13 | 134 (15.2%) | 246 (40.4%) | 0 (0.0%) | 271 (37.0%) | 651 (27.9%) |
| ≥14 | 41 (4.6%) | 43 (7.1%) | 7 (6.6%) | 49 (6.7%) | 140 (6.0%) |
| *Missing data* | *97 (11.0%)* | *106 (17.4%)* | *0 (0.0%)* | *64 (8.7%)* | *267 (11.5%)* |
| **Job condition** |  |  |  |  |  |
| Employed | 404 (45.8%) | 170 (27.9%) | 17 (16.0%) | 236 (32.2%) | 827 (35.5%) |
| Unemployed | 277 (31.4%) | 318 (52.2%) | 89 (84.0%) | 437 (59.6%) | 1,121 (48.1%) |
| Invalid | 102 (11.6%) | 4 (0.7%) | 0 (0.0%) | 0 (0.0%) | 106 (4.5%) |
| *Missing data* | *100 (11.2%)* | *117 (19.2%)* | *0 (0.0%)* | *60 (8.2%)* | *277 (11.9%)* |
| **Family arrangement ^§^** |  |  |  |  |  |
| Living with family | 686 (77.7%) | 427 (70.1%) | 25 (23.6%) | NA | 1,138 (71.2%) |
| Living in community | 55 (6.2%) | 13 (2.1%) | 4 (3.8%) | NA | 72 (4.5%) |
| Living alone | 79 (8.9%) | 63 (10.3%) | 4 (3.8%) | NA | 146 (9.1%) |
| *Missing data* | *63 (7.2%)* | *106 (17.5%)* | *73 (68.8%)* | *NA* | *242 (15.2%)* |
| **Marital status** |  |  |  |  |  |
| Unmarried | 665 (75.3%) | 436 (71.6%) | 83 (78.3%) | 577 (78.7%) | 1,761 (75.5%) |
| Married | 110 (12.5%) | 53 (8.7%) | 17 (16.0%) | 81 (11.1%) | 261 (11.2%) |
| Separated | 19 (2.2%) | 19 (3.1%) | 6 (5.7%) | 22 (3.0%) | 66 (2.8%) |
| Divorced | 11 (1.2%) | 14 (2.3%) | 0 (0.0%) | 6 (0.8%) | 31 (1.3%) |
| Widow/er | 1 (0.1%) | 1 (0.2%) | 0 (0.0%) | 0 (0.0%) | 2 (0.1%) |
| *Missing data* | *77 (8.7%)* | *86 (14.1%)* | *0 (0.0%)* | *47 (6.4%)* | *210 (9.0%)* |
| **Clinical status†** |  |  |  |  |  |
| Optimal | 604 (68.4%) | 436 (71.6%) | 79 (74.5%) | 508 (69.3%) | 1,627 (69.8%) |
| Good | 167 (18.9%) | 127 (20.9%) | 9 (8.5%) | 143 (19.5%) | 446 (19.1%) |
| Intermediate | 94 (0.6%) | 33 (5.4%) | 14 (13.2%) | 68 (9.3%) | 209 (9.0%) |
| High-intermediate | 14 (1.6%) | 7 (1.1%) | 2 (1.9%) | 9 (1.2%) | 32 (1.4%) |
| Poor | *4 (1.5%)* | *6 (1.0%)* | *2 (1.9%)* | *5 (0.7%)* | *17 (0.7%)* |

§ Information for Lazio Region was not available for this characteristic, which was calculated on the 1,598 remaining patients.

† The clinical status was assessed by the Multisource Comorbidity Score (MCS) according to the hospital admission and the drugs prescribed in the two-year period before the index date. Five categories of clinical status were considered: optimal (score=0), good (1≤score≤5), intermediate (6≤score≤10), high-intermediate (11≤score≤15) and poor (score ≥ 16).
